# Supplementary material for: Transcriptional decomposition reveals active chromatin architectures and cell specific regulatory interactions
Source: Nat Commun. 2018 Feb 5;9:487. doi: 10.1038/s41467-017-02798-1 (PMC5799294; doi:10.1038/s41467-017-02798-1)
Supplement: Supplementary file 1 — Supplementary Information [file 41467_2017_2798_MOESM1_ESM.pdf]

Supplementary material to:

Transcriptional decomposition reveals active chromatin architectures and cell specific regulatory interactions

Sarah Rennie[1], **Maria Dalby**[1], **Lucas van Duin**[1], **and Robin Andersson**[1,*]

[1]The Bioinformatics Centre, Department of Biology, University of Copenhagen, Ole Maaloes Vej 5, DK-2200, Copenhagen, Denmark
[*]Corresponding author: Robin Andersson (robin@binf.ku.dk)

Supplementary Figures

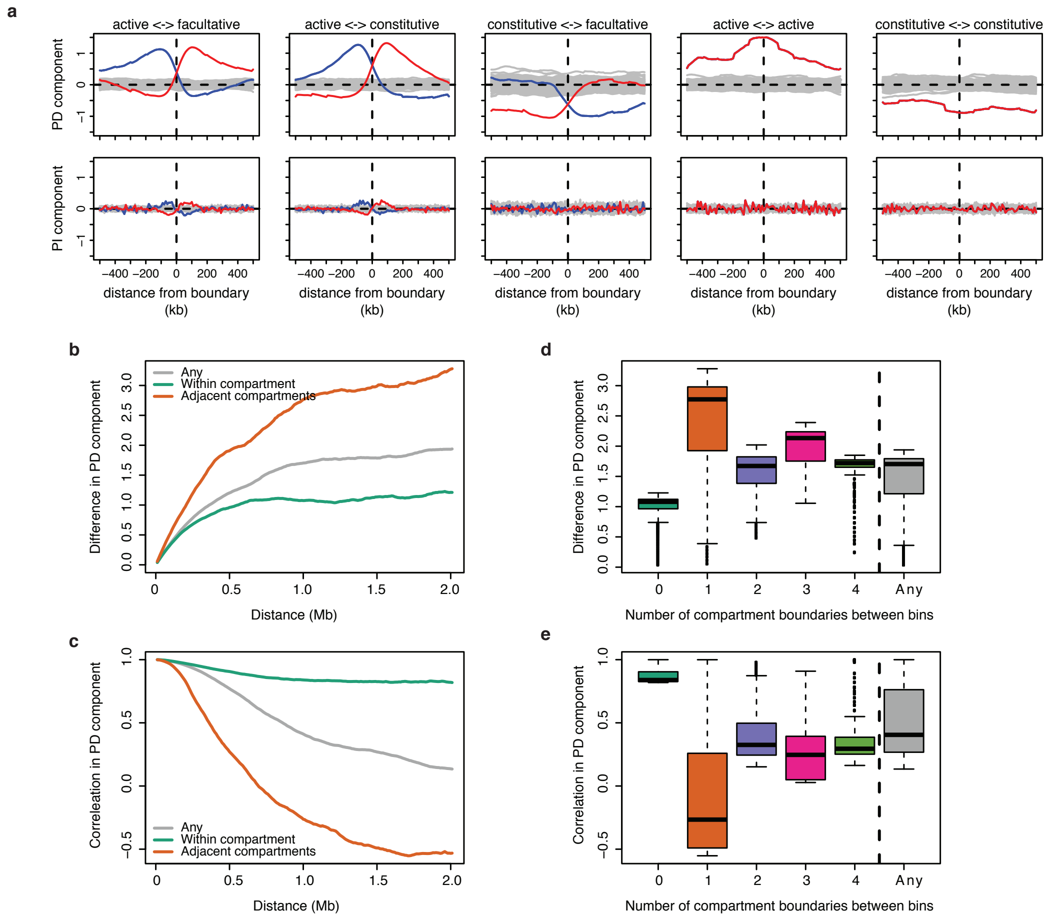

Supplementary Figure 1. Relationship between transcriptional components and compartments.
a: Average PD signal (top row) and PI signal (bottom row) around boundaries of HiC-derived chromatin compartments. Blue represents shifts from 5' to 3' genomic coordinates red represents shifts from 3' to 5'. Horizontal dotted lines represent the transition between positive and negative and grey bands represent equivalent shifts across random compartment boundaries. **b-c**: Difference in PD component (**b**) and correlation (**c**) between cell lines, plotted according to distance between bins, for bin pairs falling within the same compartment (green), across adjacent compartments (orange) and any (grey).
d-e: Box-and-whisker plots representing the distribution of differences in PD component (**d**) and correlations (**e**) across ENCODE cell lines, according to the number of compartments spanned by bin-pairs (up to 4 apart, and any). The lower and upper hinges of boxes correspond to the first and third quartiles of data, respectively, and the whiskers extends to the largest and smallest data points no further away than 1.5 times the interquartile range.

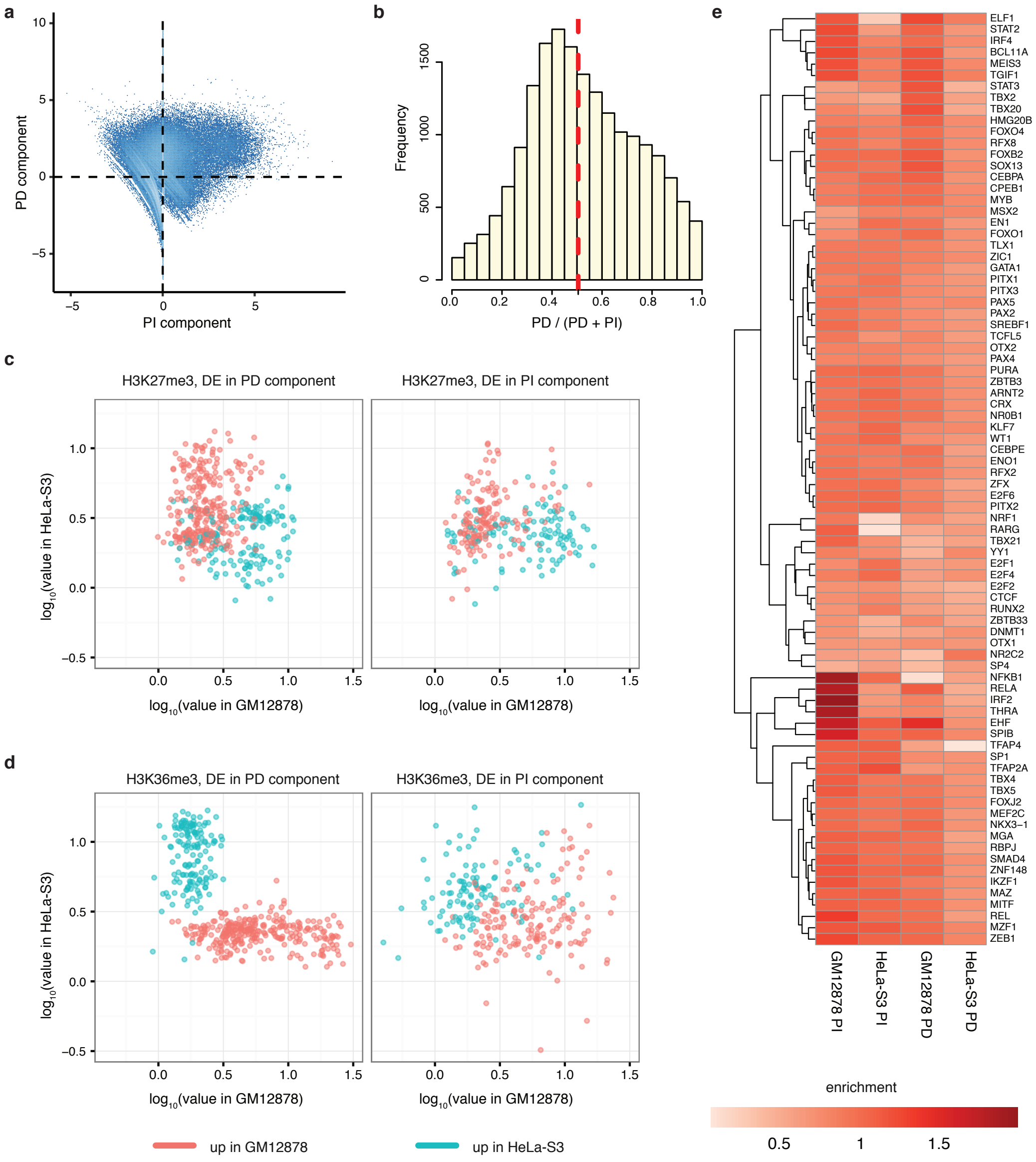

Supplementary Figure 2. Separability of PD and PI components. a: Scatter plot of the PD component against the PI component in GM12878. Dotted lines represent the points where the components are equal to zero. **b**: Histogram of the ratio of the PD component to the sum of the PD and PI components, for bins containing expressed TUs. The frequency represents the number of TUs and the red dashed line represents the median. **c**: Differences in H3K27me3 levels (TPM normalised and aggregated bin-wise) between HeLa-S3 (vertical axes) and GM12878 (horizontal axes) cells at differentially expressed (DE) bins in the PD (left) and PI (right) components up in GM12878 (red) or up in HeLa-S3 (green). **d**: Differences in H3K36me3 levels (TPM normalised and aggregated bin-wise) between HeLa-S3 (vertical axes) and GM12878 (horizontal axes) cells at DE bins in the PD (left) and PI (right) components up in GM12878 (red) or up in HeLa-S3 (green). **e**: Expressed TF motif enrichment around expressed CAGE-derived promoters associated with GM12878 or HeLa-S3 biased differentially expressed PD or PI bins.

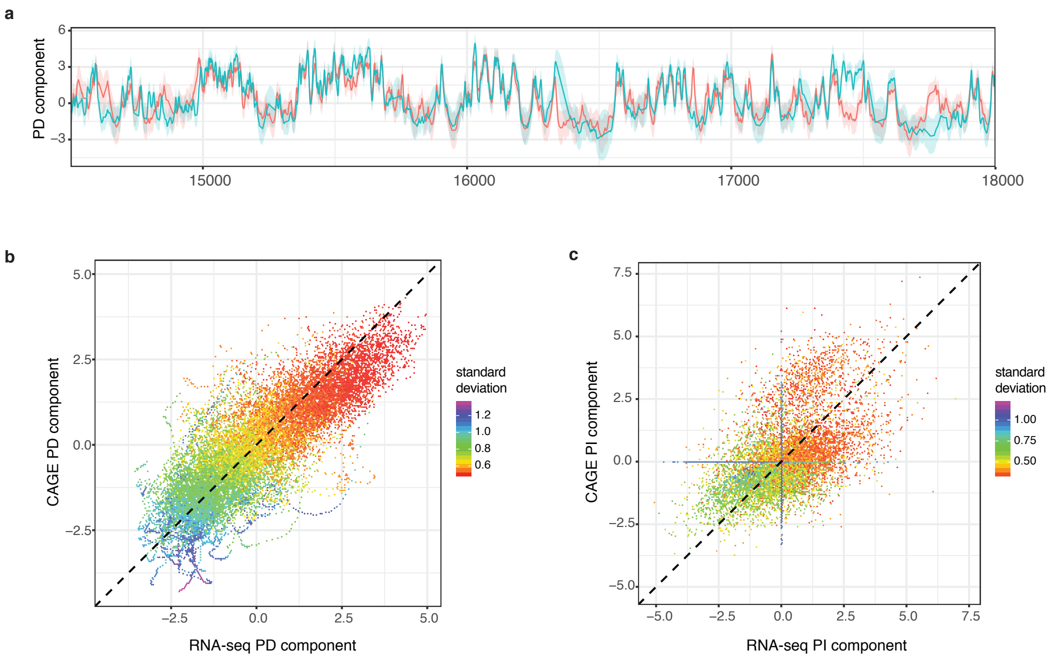

Supplementary Figure 3. Transcriptional decomposition performed using RNA-seq data. a: PD components in GM12878 for CAGE (red) and RNA-seq (green), plotted (+/-) their respective standard deviations around their estimated posterior values. Locus plotted is chr1:145,000,000-180,000,000. **b-c**: Scatter plots of the PD component (**b**) and the PI component (**c**) for CAGE against RNA-seq, for all 10kb bins on chromosome 1 within 25 bins of an active TU. Colour scheme is generated according to the sums of the standard deviations from the two datasets.

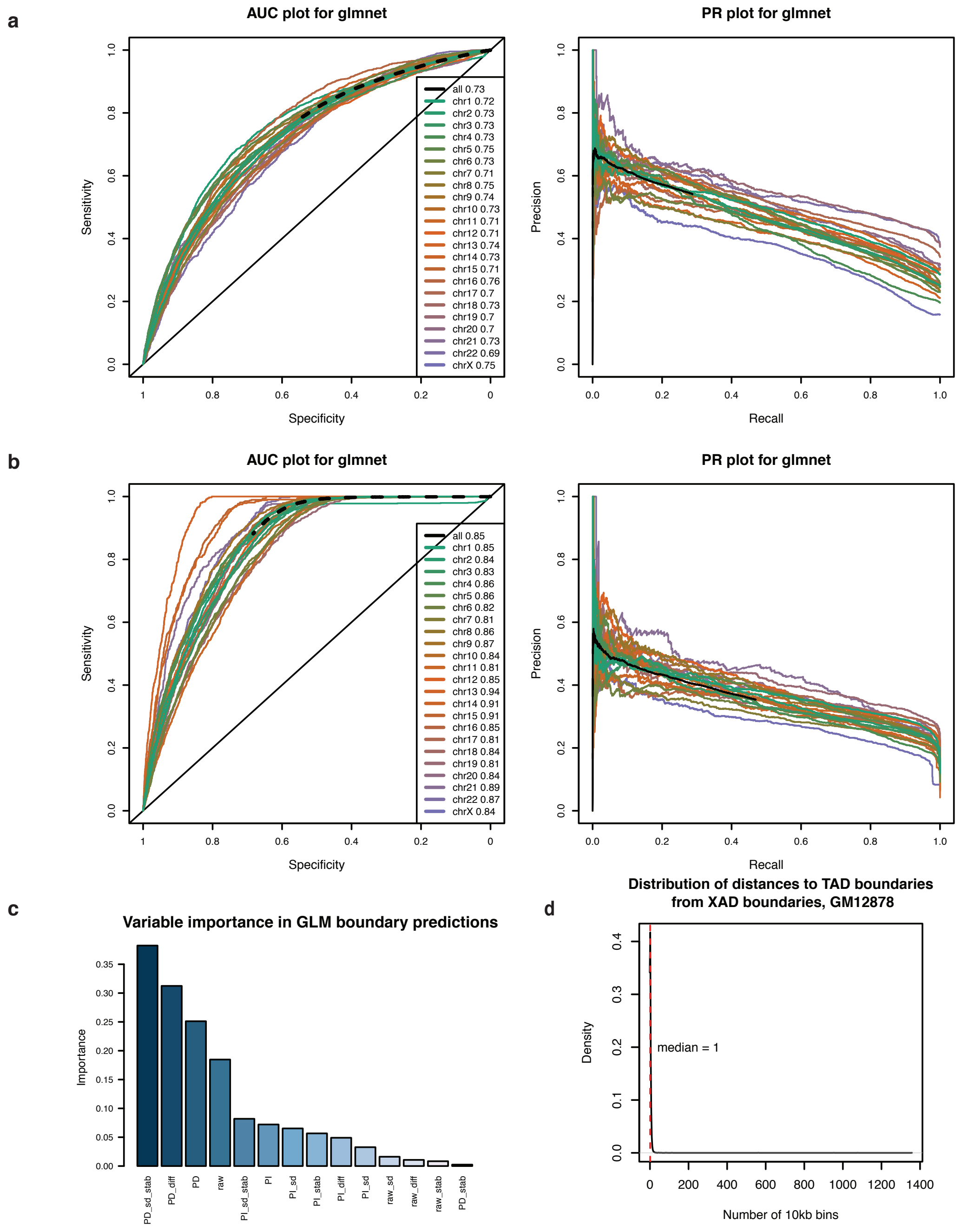

Supplementary Figure 4. Predictability of TAD boundary regions from transcriptional components. a-b: ROC and precision-recall (PR) curves for glmnet model, predicting all TAD boundary regions (**a**) and restricting on TAD boundary regions within positive PD regions (**b**). Performance based on held out data based on 2-fold cross validation and generated for the whole dataset as well as per chromosome. **c**: Relative importance of variables from the generalised linear model fit. **d**: Density plot showing the distribution of distances of TAD boundaries in GM12878 from XAD boundaries. Red dashed line indicates the median of a single 10kb bin from a TAD boundary.

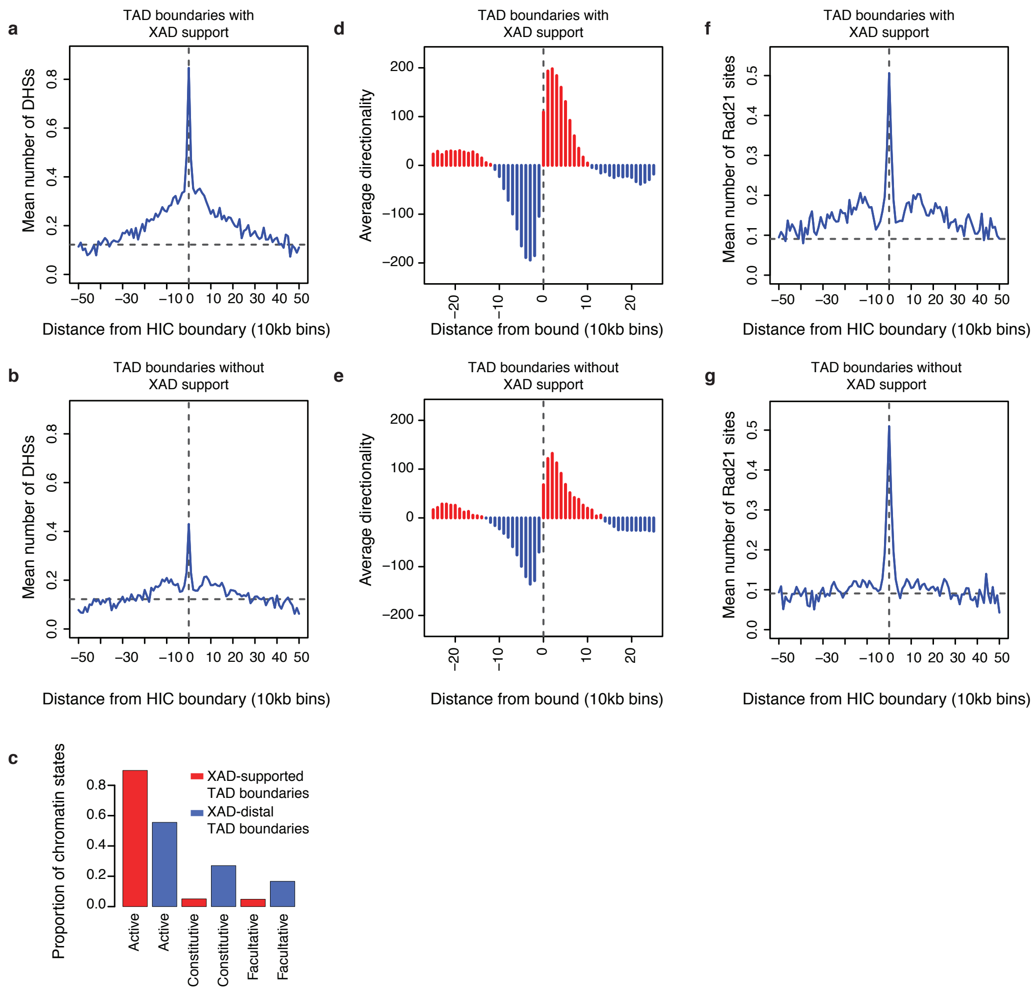

Supplementary Figure 5. Properties of TAD boundaries according to XAD boundary support. **a-b**: Enrichment of DHSs around TAD boundaries when supported by XAD boundaries (within +/-5 bins) (**a**) and when unsupported by XAD boundaries (**b**). **c**: Proportion of TAD boundaries within annotated chromatin compartments, according to XAD boundary support. **d-e**: HiC directionality score around TAD boundaries when supported by XAD boundaries (within +/-5 bins) (**d**) and when unsupported by XAD boundaries (**e**). **f-g**: Enrichment of Rad21 binding sites around TAD boundaries when supported by XAD boundaries (within +/-5 bins) (**f**) and when unsupported by XAD boundaries (**g**).

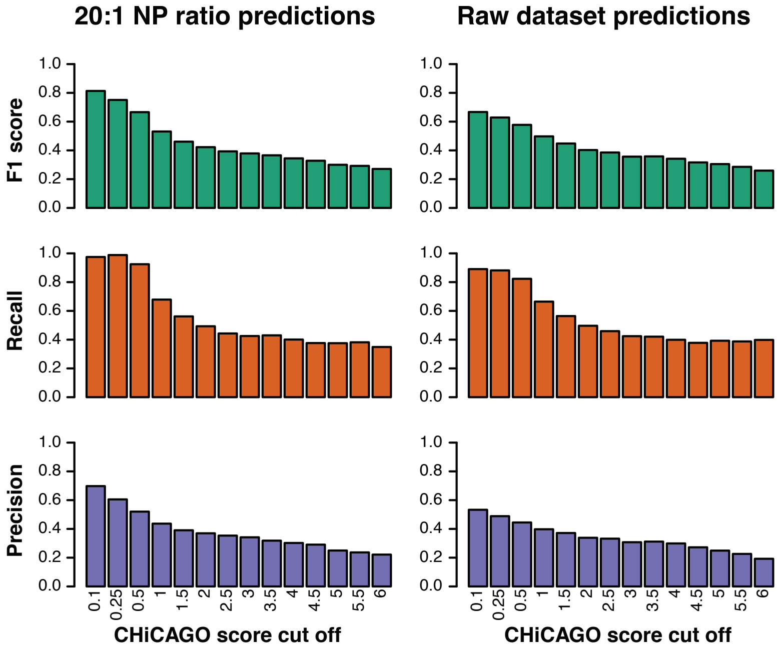

Supplementary Figure 6. Effect of CHiCAGO score threshold on EP interaction prediction performance. F1 score (top), recall (middle), and precision (bottom) of models for EP interactions, according to different CHiCAGO scores (horizontal axes), when evaluated at a 20:1 negative-to-positive (NP) ratio (left) and at raw NP ratio (right). Models are based on training on a lenient CHiCAGO score cut off ($\geq$3) and probability cut offs are based on F1 efficient cut-offs (distance variant) for each performance score threshold.

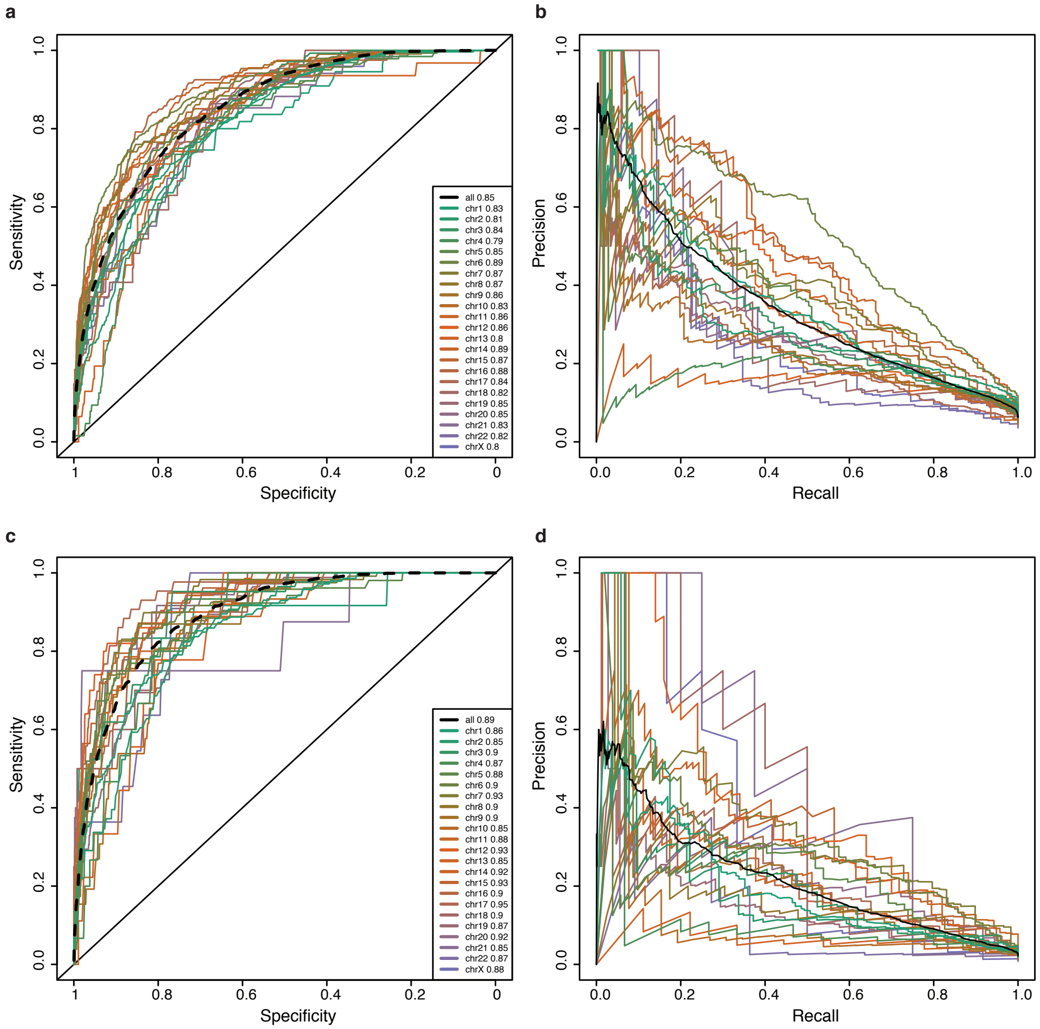

Supplementary Figure 7. Global model performance for EP interactions, per chromosome. a-d: Chromosome specific ROC curves (**a,c**) and precision-recall curves (**b,d**) for performance in predicting EP interactions in GM12878 cells, for CHiCAGO score ≥ 3 (**a,b**), or CHiCAGO score ≥ 5 (**c,d**). Black dashed lines represents performance across all chromosomes together.

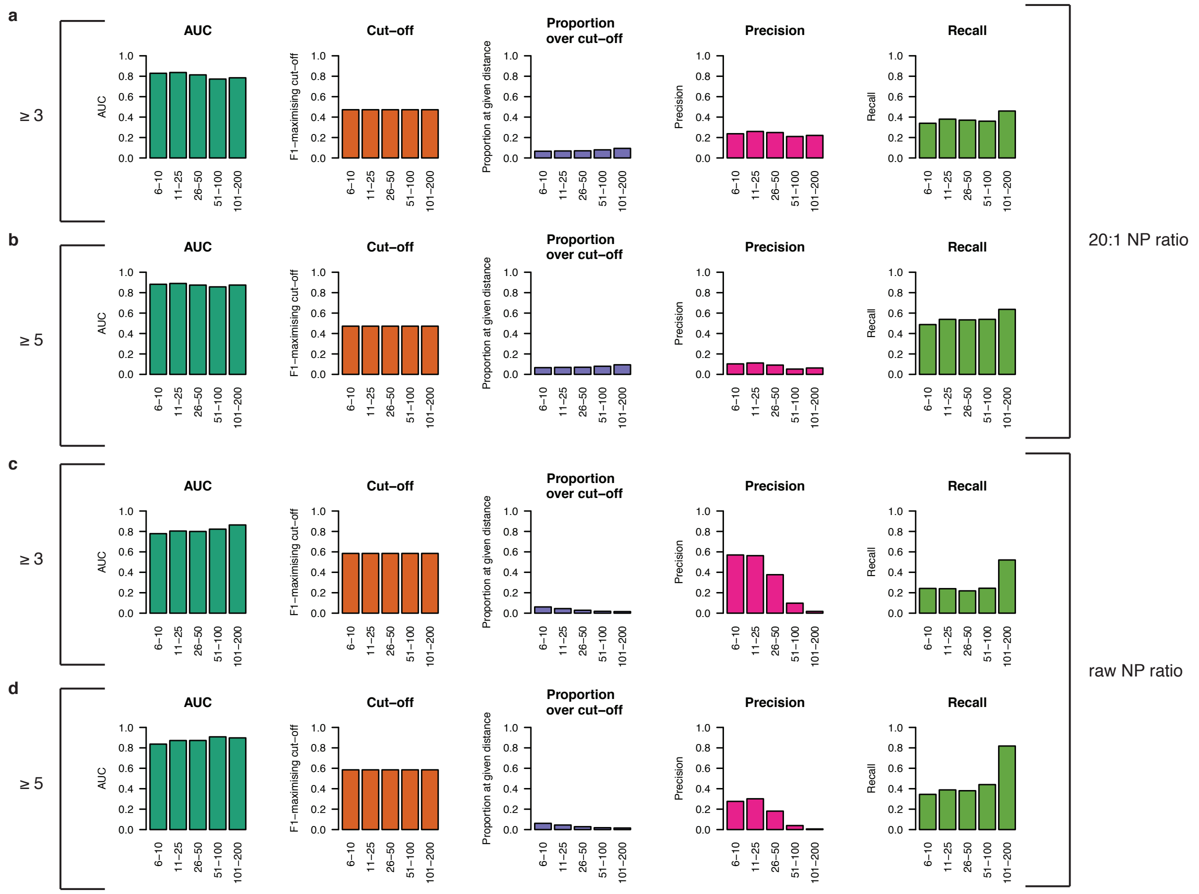

Supplementary Figure 8. Model performance for EP interactions for a fixed cut-off over distance in GM12878. Fixed probability cut-off based on maximising the F1 statistic and performance statistics supplied separately over 5 distance groups. Results are given for both the 20:1 NP ratio test dataset (**a,b**) and the raw NP ratio test dataset (**c,d**), and according to CHiCAGO score ≥ 3 (**a,c**) and CHiCAGO score ≥ 5 (**b,d**).

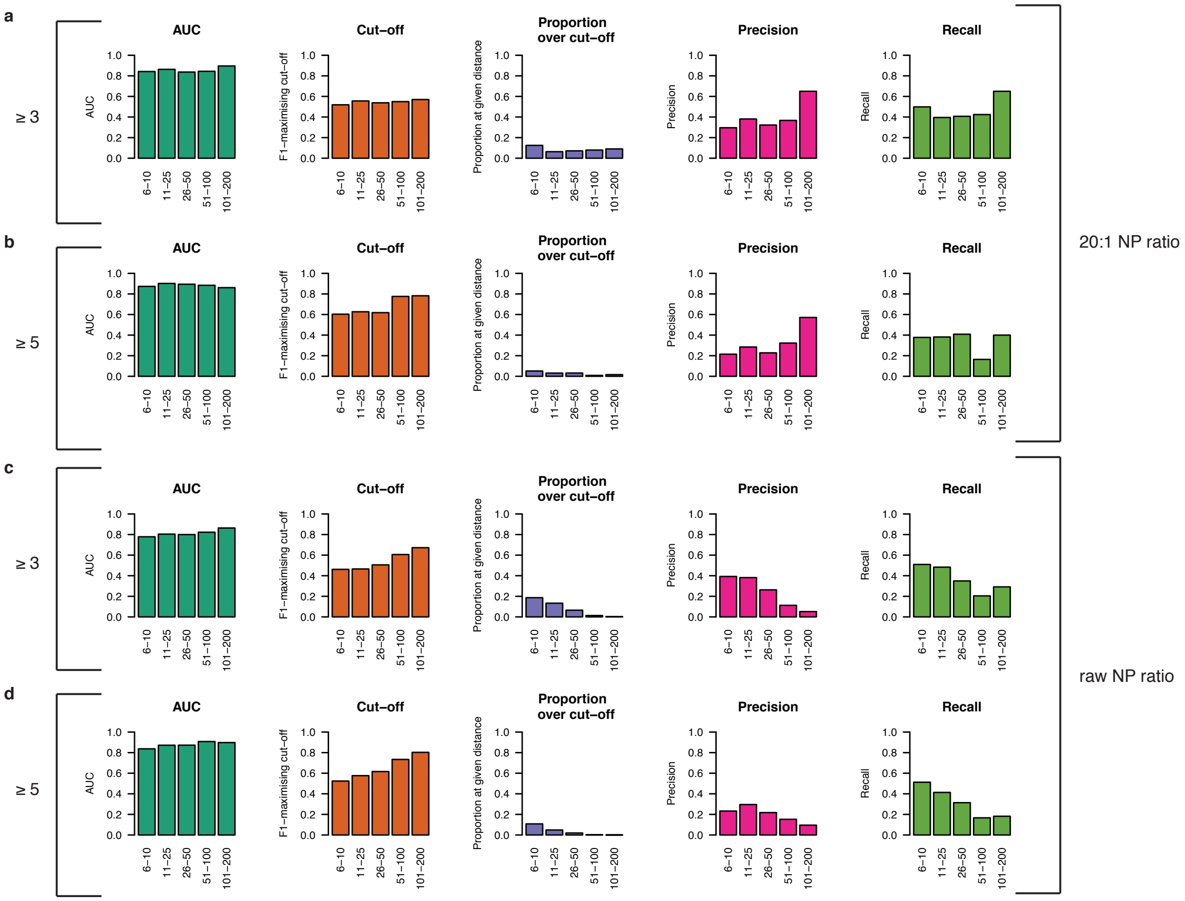

Supplementary Figure 9. Model performance for EP interactions for varying cut-offs over distance in GM12878. Data split over 5 distance thresholds and cut-off chosen to be the F1 maximising cut-off at each threshold. Results are given for both the 20:1 NP ratio test dataset (**a,b**) and the raw NP ratio test dataset (**c,d**), and according to CHiCAGO score ≥ 3 (**a,c**) and CHiCAGO score ≥ 5 (**b,d**).

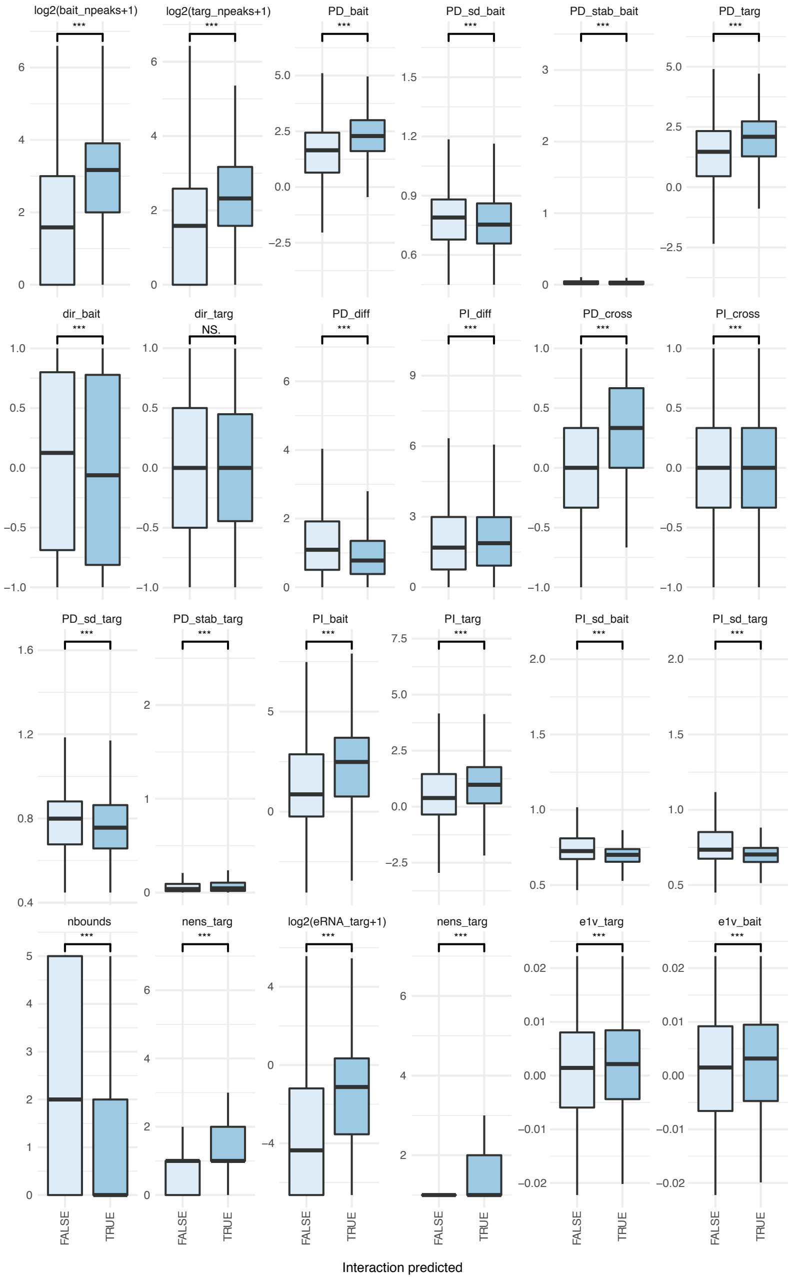

Supplementary Figure 10. Features separating predicted interacting versus predicted non-interacting enhancer-promoter pairs.. Box and whisker plots for transcriptional based features, split according to predicted (darker blue, TRUE) versus non-predicted (light blue, FALSE) enhancer-promoter interactions in GM12878. Significance based on pair-wise t-tests such that * = FDR<0.05, ** = FDR<0.01, *** = FDR<0.001 and NS = non-significant. Outliers are not included in the box and whisker plots.

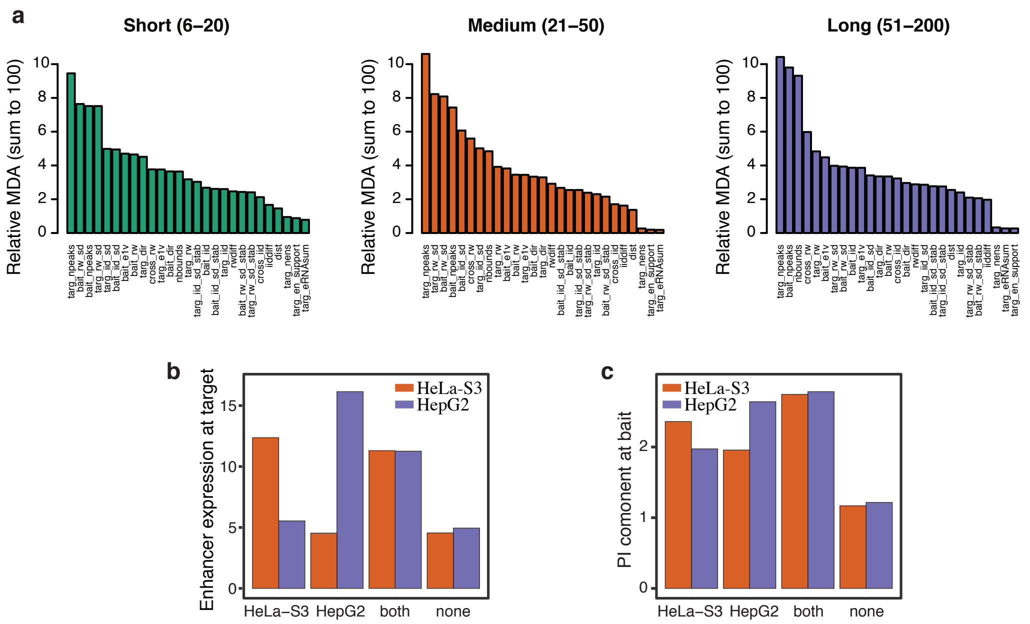

Supplementary Figure 11. Feature importances for predicting enhancer-promoter interactions. **a**: Feature importance across three different sets of distances. **b-c**: Enhancer transcription at the target (**b**) and PI component at the bait (**c**) at cell-type specific, shared, and non-predicted promoter-enhancer interactions. Bars represent mean feature levels for HeLa-S3 and HepG2 cells according to whether the interaction was predicted in HeLa-S3 only, HepG2 only, both, or none.

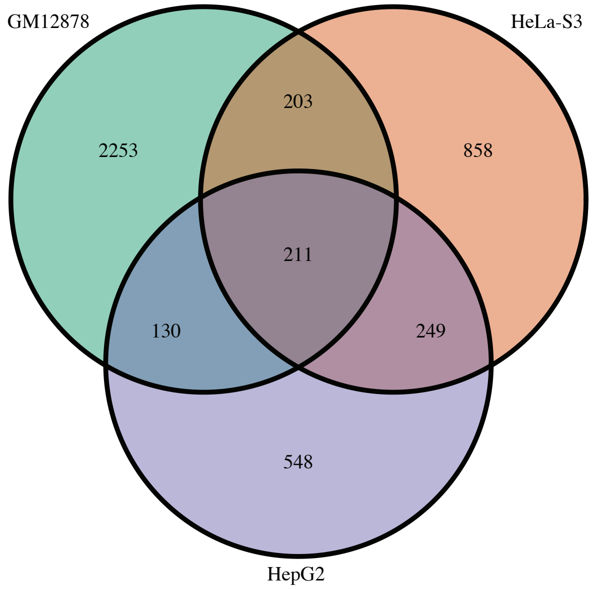

Supplementary Figure 12. Effect of CHiCAGO score threshold on cell type differences in predicted EP interactions. Number of EP interactions shared in GM12878, HeLa-S3 and HepG2, for CHiCAGO score ≥ 5. Prediction cut off set by maximising the F1 statistic (distance variant) in GM12878.

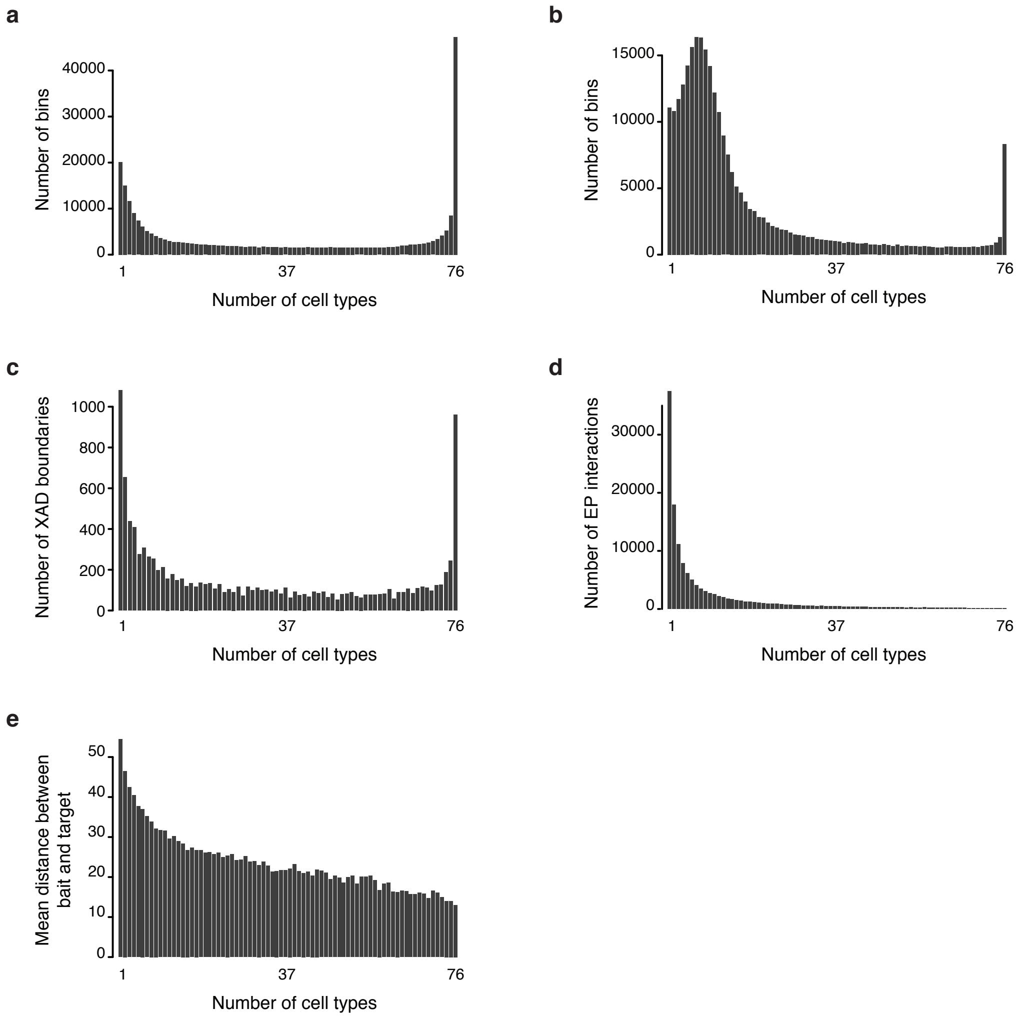

Supplementary Figure 13. Similarities between cell types in transcriptional components. a: Sharing of bins with positive PD signal across cell types. **b**: Sharing of bins with positive PI signal across cell types. **c**: Sharing of XAD boundary regions across cell types at 100kb resolution. **d**: Sharing of predicted EP interactions across cell types. For all interactions present in at least one cell type, bars represent the number of cell types in which it was predicted. **e**: Sharing of predicted EP interactions across cell types versus mean distance between bait (promoter) and target (enhancer).

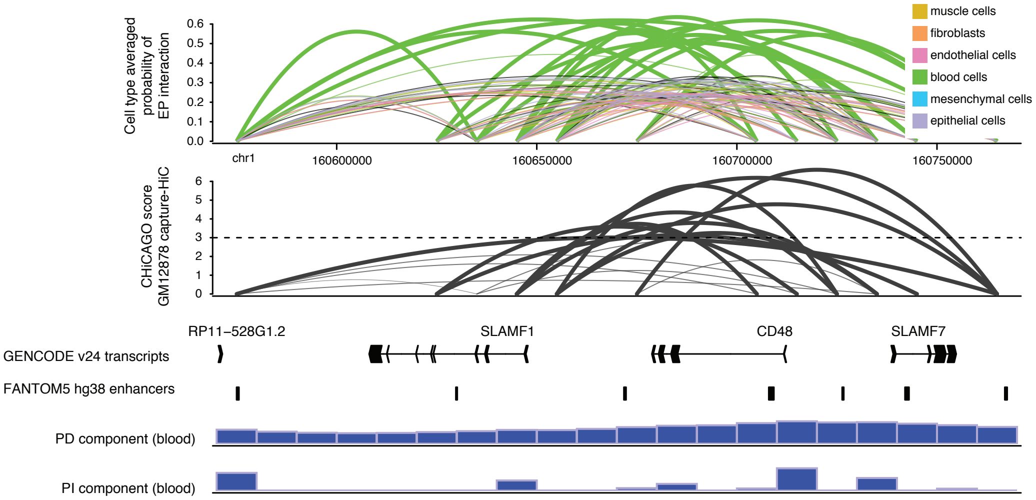

Supplementary Figure 14. Transcriptional decomposition reveals cell-type specific regulatory organisation around *CD48* gene. From top to bottom: predicted probability of EP interactions averaged across groups of cells (as in Figure 6d), CHiCAGO score of interaction based on GM12878 capture HiC data. Below are displayed (in the following order) GENCODE v24 transcripts, FANTOM5 enhancers, the average PD and PI component across blood cells.

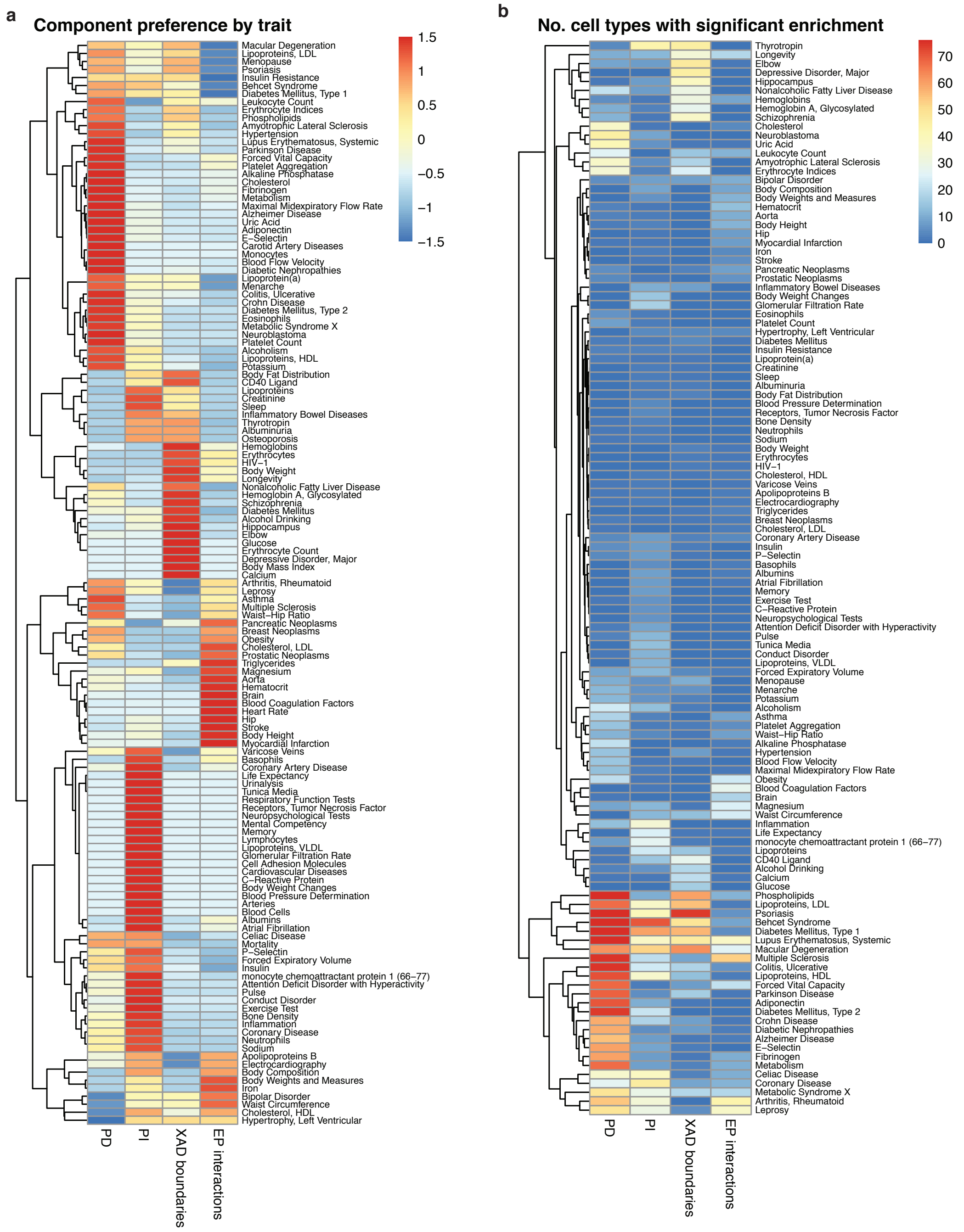

Supplementary Figure 15. Component and cell type preferences of GWAS trait SNP enrichments. **a**: Preferences of GWAS trait-associated SNPs in PD positive bins, PI positive bins, XAD boundaries and EP interactions identified in 76 human cell types. Preference calculated based on the number of significant (χ^2 test, FDR<0.01 and odds>1.25) cell types per trait and component (see methods), scaled across the four components. **b**: Number of cell types with significant (as in **a**) enrichments of GWAS trait-associated SNPs in PD positive bins, PI positive bins, XAD boundaries and EP interactions identified in 76 human cell types.

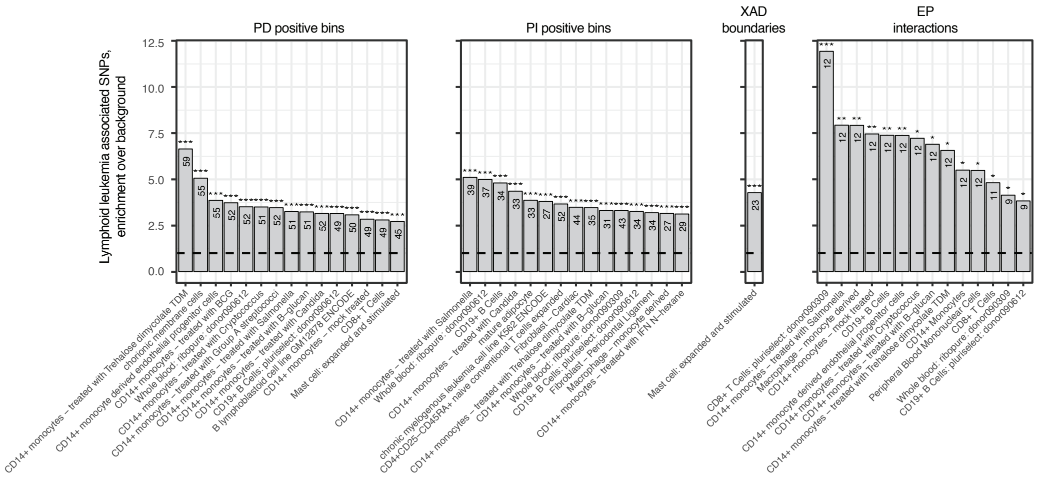

Supplementary Figure 16. Analysis of lymphoid leukemia associated SNPs reveals cell-type and expression component preferences. Lymphoid leukemia SNP enrichments in transcriptional components, XAD boundaries, and inferred enhancer-promoter interactions (FDR-corrected χ^2 tests based on the PD/PI positive bins or presence of XAD boundaries/target enhancers per cell type, and trait associated SNPs from the GWAS catalog, see Methods). Significance stars above bars are interpreted as: $* = P < 0.1$, $** = P < 0.01$ or $*** = P < 0.001$.

Supplementary Tables

Supplementary Table 1. Best correlation parameter combinations for RNA-seq transcriptional decomposition, per chromosome. INLA hyperparameters (size and probability parameters of the negative binomial distribution, precision for the PD component (RW), precision for the PI component (IID)) for RNA-Seq data, which were found to give the best correlation to the PD components of CAGE data. Both RNA-Seq data and CAGE data derived from GM12878 cells.

Chromosome	size	prob	RW	IID	PD correlation	PI correlation
1	8	-1	-4	2	0.8343338	0.8685478
2	8	-1	-7	-1	0.8314016	0.8384131
3	8	-4	-4	2	0.8426355	0.8464501
4	8	3	-4	2	0.8047164	0.7977146
5	8	-4	-4	2	0.8168530	0.8325121
6	8	-4	-4	2	0.8068548	0.8185471
7	8	-4	-6	-1	0.8285529	0.8204263
8	8	-4	-4	2	0.8088375	0.8117918
9	2	3	-4	2	0.8329053	0.9064663
10	7	-4	-4	2	0.8109703	0.8298349
11	2	-4	-4	2	0.8575597	0.8587555
12	2	-4	-4	2	0.8514417	0.8623694
13	7	3	-3	2	0.8114826	0.9815640
14	8	2	-3	2	0.7948931	0.9673565
15	8	-4	-3	2	0.8277752	0.9636634
16	2	2	-3	2	0.8642273	0.8265529
17	2	-4	-3	2	0.8772700	0.8866936
18	1	3	-6	-1	0.7759199	0.7527304
19	2	-4	-3	2	0.8981290	0.9118324
20	7	-4	-3	2	0.8327296	0.8354974
21	1	-3	-5	-1	0.7631566	0.9470045
22	7	2	-5	-1	0.8484782	0.9509241
X	7	3	-3	2	0.8150549	0.8073811

Supplementary Table 2. Features used for TAD GLM training. PI and PD features based on chromosomal transcriptional decomposition model for GM12878. Abbrev. gives the abbreviated names for the features and Description explains how the features were derived.

Feature	Abbrev.	Description
PD signal	PD	Posterior PD estimate
PD standard deviation	PD_sd	Posterior PD standard deviation
PD stability	PD_stab	Standard deviation across cell types of PD
PD sd stability	PD_sd_stab	Standard deviation across cell types of PD_sd
PD difference	PD_diff	First order difference of PD
PI signal	PI	Posterior PI estimate
PI standard deviation	PI_sd	Posterior PI standard deviation
PI stability	PI_stab	Standard deviation across cell types of PI
PI sd stability	PI_sd_stab	Standard deviation across cell types of PI_sd
PI difference	PI_diff	First order difference of PI
RAW signal	raw	Mean $\log 2$ TPM over replicates
RAW difference	raw_diff	First order difference of raw
RAW standard deviation	raw_sd	Standard deviation of $\log 2$ TPM over replicates
RAW stability	raw_stab	sd across cell types of mean $\log 2$ TPM over replicates

Supplementary Table 3. List of features trained against significant proximity interactions. Features calculated for the bait and/or the target separately, or using information from both. Features calculated based using information from all cell types are listed as non-cell type specific (N), in contrast to those which may be calculated only from the cell type of interest (Y). Data type listed as continuous (C) or discrete (D), whereby the numbers in brackets indicate the possible values which the feature may take.

Term	Abbreviation(s)	Cell-type specific?	Data type
PD value at bait/target	PD_bait,PD_targ	Y	C
PD sd value at bait/target	PD_sd_bait PD_sd_targ	Y	C
PD stability at bait/target	PD_stab_bait,PD_stab_targ	N	C
PI value at bait/target	PI_bait,PI_targ	Y	C
PI sd value	PI_sd_bait,PI_sd_targ	Y	C
PD difference	PD_diff	Y	C
PI difference	PI_diff	Y	C
PD cross correlation	PD_cross	N	C
PI cross correlation	PI_cross	N	C
Enhancer expression at target	eRNA_targ	Y	C
Directionality at bait/target	dir_bait, dir_targ	Y	C
First eigenvector of PD cross correlation	e1v_bait, e1v_targ	N	C
XAD boundary insulation	nbounds	Y	D (0-3)
Distance between bins	dist	N	D (6-200)
Peaks detected at bait/target	npeaks_bait,npeaks_targ	Y	D (0+)
Predicted enhancers at target	nens_targ	Y	D (0+)
No. cell lines supporting target enhancer	en_support_targ	N	D (0+)

Supplementary Table 4. **Number of positives called from the promoter-capture HiC GM12878 data, ENCODE cell line datasets set.** ENCODE peak refers to a peak transcribed in more than one replicate in at least one of the ENCODE datasets. Enhancers refers to a CAGE defined enhancer which is transcribed in at least one of the ENCODE datasets.

Bait	Target	Total # pairs	CHiCAGO score	# positive pairs
CHIC baits+ENCODE peak	ENCODE peak	1,832,906	$\geq$3	41,138
CHIC baits+ENCODE peak	ENCODE peak	1,832,906	$\geq$5	11,603
CHIC baits+ENCODE peak	Enhancers	197,394	$\geq$3	4,723
CHIC baits+ENCODE peak	Enhancers	197,394	$\geq$5	1,447

Supplementary Table 5. **Number of positives called from the promoter-capture HiC GM12878 data, 76 datasets set.** Any peak refers to a peak transcribed in more than one replicate in at least one of the 76 datasets. Enhancers refers to a CAGE defined enhancer which is transcribed in at least one of the 76 datasets.

Bait	Target	Total # pairs	CHiCAGO score	# positive pairs
CHIC baits+Any peak	Any peak	4,012,550	$\geq$3	71,044
CHIC baits+Any peak	Any peak	4,012,550	$\geq$5	18,495
CHIC baits+Any peak	Enhancers	863,039	$\geq$3	16,989
CHIC baits+Any peak	Enhancers	863,039	$\geq$5	4,617
