# Supplementary material for: Transcriptional decomposition reveals active chromatin architectures and cell specific regulatory interactions
Source: Nat Commun. 2018 Feb 5;9:487. doi: 10.1038/s41467-017-02798-1 (PMC5799294; doi:10.1038/s41467-017-02798-1)
Supplement: Supplementary file 3 — Description of Additional Supplementary Files [file 41467_2017_2798_MOESM3_ESM.pdf]

Description of Additional Supplementary Files

File Name: Supplementary Data 1
Description: Differentially expressed bins in the PD component. Based on posterior estimates of the difference between bins in GM12878 versus HeLa-S3. Bins with FDR<0.01, corrected according to the number of raw-expressed bins, are considered significant. Score listed as 1 if the bin PD component was significant up in HeLa-S3 compared to GM12878, and -1 if up in GM12878 compared to HeLa-S3.

File Name: Supplementary Data 2
Description: Differentially expressed bins in the PI component. Based on posterior estimates of the difference between bins in GM12878 versus HeLa-S3. Bins with FDR<0.01, corrected according to the number of raw-expressed bins, are considered significant. Score listed as 1 if the bin PD component was significant up in HeLa-S3 compared to GM12878, and -1 if up in GM12878 compared to HeLa-S3.

File Name: Supplementary Data 3
Description: Predicted GM12878 EP interactions. Bin set based on intra-chromosomal enhancers and baits active across ENCODE cell lines. Predictions derived from 10-fold validation testing on model training on GM12878 CaptureHIC CHICAGO score-derived significant interactions. EP interactions predicted for score cut-off ≥ 3 and ≥ 5. See Methods for further details.

File Name: Supplementary Data 4
Description: Predicted HeLa-S3 EP interactions. Bin set based on intra-chromosomal enhancers and baits active across ENCODE cell lines. Predictions derived from testing HeLa-S3 features from model training on GM12878 CaptureHIC CHICAGO score-derived significant interactions. EP interactions predicted for most efficient cut-off based on score ≥ 3 and score ≥ 5. See Methods for further details.

File Name: Supplementary Data 5
Description: Predicted HepG2 EP interactions. Bin set based on intra-chromosomal enhancers and baits active across ENCODE cell lines. Predictions derived from testing HepG2 features from model training on GM12878 CaptureHIC CHICAGO score-derived significant interactions. EP interactions predicted for most efficient cut-off based on score ≥ 3 and score ≥ 5. See Methods for further details.

File Name: Supplementary Data 6
Description: Replicated CAGE samples and associated FANTOM5 ids used in the analysis.
